# Supplementary material for: Immunostimulatory Effects of Polysaccharides from Spirulina platensis In Vivo and Vitro and Their Activation Mechanism on RAW246.7 Macrophages
Source: Mar Drugs. 2020 Oct 28;18(11):538. doi: 10.3390/md18110538 (PMC7692637; doi:10.3390/md18110538)
Supplement: Supplementary file 1 [file marinedrugs-18-00538-s001.pdf]

Supplementary Information

S1. Determination of homogeneity and molecular weight

The molecular weight of PSP-1, PSP-2 and PSP-3 was determined through high performance gel filtration chromatography technique (HPGFC) using an HPLC system that was equipped with a TSK-gel G3000PW column (7.8 mm×300 mm) and an evaporative light scattering detector (Alltech, USA). The polysaccharide solution (2 mg/mL) was dissolved in 50 mmol/L ammonium acetate. After filtration through a 0.45 μm of membrane, the 10 μL filtrate was injected into the HPLC system. The column was eluted with 50 mmol/L ammonium acetate at a flow rate of 0.8 mL/min. Dextran standards (200 kDa, 70 kDa, 40 kDa, 10 kDa, 5 kDa) for GPC were used, and a calibration curve was drawn.

3 kinds of PSP exhibited multiple peaks in the HPLC chromatograms, as shown in Figure S-1, indicating that they were inhomogenous polysaccharides. On the basis of the equation derived from the standard curve, the molecular weights of different peaks of PSPs were calculated and the results were shown in Table S-1. PSP-1 is mainly concentrated in 49.7kDa and 20.7kDa, PSP-2 concentrated in 32.9kDa, 8.0kDa, 20 KDa and 49.7kDa, and PSP-3 concentrated in 8.0kDa, 32.9kDa, 20.1kDa and 1.0 kDa.

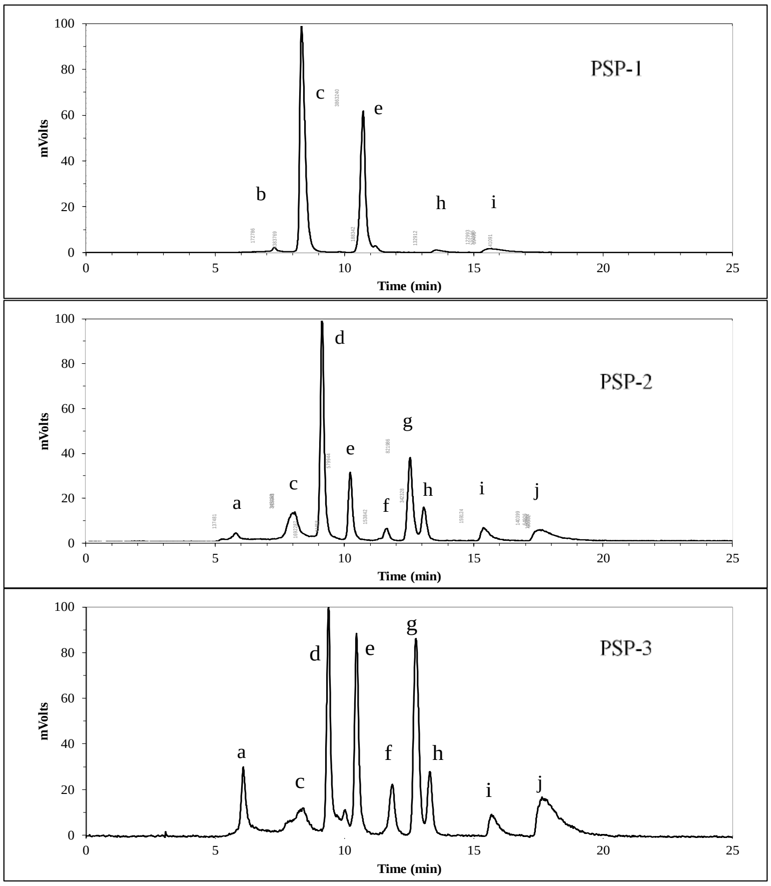

Figure S-1 HPLC elution of polysaccharide PSP-1, PSP-2 and PSP-3

Table S-1 the ratio of HPLC peaks area of PSP

	a	b	c	d	e	f	g	h	i	j
Time (min)	6.12	7.31	8.37	9.35	10.45	11.32	12.71	13.24	15.65	17.6
Mw(kDa)	129.7	78.1	49.7	32.9	20.7	14.3	8.0	6.4	2.3	1.0
PSP-1		0.59	59.28		37.79			0.77	1.57	
PSP-2	1.26		11.24	37.84	12.71	2.61	17.37	5.79	4.24	6.94
PSP-3	6.53		6.61	17.93	16.91	6.18	20.75	5.23	4.05	15.81

S2. Determination of sulfate content and uronic acid

Sulfate content was analyzed with barium chloride–gelatin method of Kawai et al. [1]. Uronic acid was estimated in a modified carbazole method using d-glucuronic acid as standard [2]. The result was shown in Table S-2.

Table S-2 Sulfate and uronic acid content of PSP-1, PSP-2 and PSP-3

	PSP-1	PSP-2	PSP-3
Sulfate content	0.06±0.007%	16.82±1.31%	17.62±1.05%
Uronic acid	3.65%±0.15	13.62%±1.02	32.68%±2.68

S3. The solubility measurement

All PSPs were incomplete dissolved. In order to evaluate the immunostimulatory effect of PSPs, their solubility was measured by dissolving 2mg PSPs into 1mL distilled water, after blending well and centrifuging, the polysaccharide content in solution was determined by phenol-sulfuric acid method. The soluble concentration of PSPs was shown in Table S-3. And all cell assays were carried out in the PSPs concentration as following.

Table S-3 The Soluble concentration of PSPs

Polysaccharide	PSP	PSP-1	PSP-2	PSP-3	PSP-H	PSP-L

Solubility (μg/mL)	315±17	429±15	216±13	155±9	189±12	361±11

1. Kawai, Y.; Seno, N.; Anno, K., A modified method for chondrosulfatase assay. *Analytical Biochemistry* 1969**,** 32, (2), 314-321.

2. Bitter, T.; Muir, H. M., A modified uronic acid carbazole reaction. *Analytical Biochemistry* 1962**,** 4, (4), 330-334.
